# A clear bias in parental origin of *de novo* pathogenic CNVs related to intellectual disability, developmental delay and multiple congenital anomalies

**DOI:** 10.1038/srep44446

**Published:** 2017-03-21

**Authors:** Ruiyu Ma, Linbei Deng, Yan Xia, Xianda Wei, Yingxi Cao, Ruolan Guo, Rui Zhang, Jing Guo, Desheng Liang, Lingqian Wu

**Affiliations:** 1 State Key Laboratory of Medical Genetics, Central South University, Changsha, Hunan 410078, P.R. China

## Abstract

Copy number variation (CNV) is of great significance in human evolution and disorders. Through tracing the parent-of-origin of *de nov*o pathogenic CNVs, we are expected to investigate the relative contributions of germline genomic stability on reproductive health. In our study, short tandem repeat (STR) and single nucleotide polymorphism (SNP) were used to determine the parent-of-origin of 87 *de novo* pathogenic CNVs found in unrelated patients with intellectual disability (ID), developmental delay (DD) and multiple congenital anomalies (MCA). The results shown that there was a significant difference on the distribution of the parent-of-origin for different CNVs types (Chi-square test, p = 4.914 × 10^−3^). An apparently paternal bias existed in deletion CNVs and a maternal bias in duplication CNVs, indicating that the relative contribution of paternal germline variations is greater than that of maternal to the origin of deletions, and vice versa to the origin of duplications. By analyzing the sequences flanking the breakpoints, we also confirmed that non-allelic homologous recombination (NAHR) served as the major mechanism for the formation of recurrent CNVs whereas non-SDs-based mechanisms played a part in generating rare non-recurrent CNVs and might relate to the paternal germline bias in deletion CNVs.

Germ cell, as the carrier of genetic information, is essential for human reproductive fitness and the development of offsprings. The health of germ cells is reflected not only in the morphology, structure and physiological function, but also in the integrity and stability of the genome they carry, which is indispensable for the constant transfer of genetic information. As a fact, during the whole life of germ cells, a wide variety of genomic variations will emerge, possibly affecting the stability of the genome. In terms of hominid evolution, these variations, including base mutations, variations of microsatellites and structural variations (SVs), play an important role in the biological diversity and the genome variability among individuals^1^,^2^,^3^. On the other hand, they are also recognized as significant contributors to the occurrence of human diseases.

CNVs, which are known as deletions or duplications of genomic materials that are larger than 1 kb in size in comparison with the reference genome^4^, have become a popular hotspot of the genomic SVs in the past decade. Many researches have reported about the close relationship between CNVs and complex genomic disorders such as ID, autism, schizophrenia and numerous developmental diseases^5^,^6^,^7^,^8^. And most of the known pathogenic and high-risk variants are *de novo*. SNP, with allele frequency at least 1%, is also one important type of variations throughout the genome. Comparatively, because of the larger size and more genetic materials involved, CNVs naturally will be more complicated. Nonetheless, whether *de novo* CNVs are more significant and abundant than SNPs in disease causation has already become a wide debate in recent years. Previous studies had emphasized that the proportion of nucleotide content in CNVs is far higher than that of SNPs per genome^9^,^10^. S.W. Scherer *et al*.^11^ found that CNVs accounted for 1.28% of genetic variants between the first single individual genome (J Craig Venter’s DNA named HuRef)^12^ and the National Center for Biotechnology Information (NCBI) reference genome assembly, whereas SNPs encompassed only 0.1%. All these underscored one significant source of genetic variations in germ cell genome—CNVs.

Nowadays, it is widely shared that CNVs derived from a variety of ways during the formation of germ cells, such as DNA repair, replication errors, homologous recombination and chromosome separation errors. The generation mechanism underlying these pathogenic CNVs has been studied extensively. Yet, the relationship between the genesis of these CNVs and their parental origin is still rarely known. Here, we launched a research on the parent-of-origin of *de novo* pathogenic CNVs found in Chinese patients with ID, DD and MCA. Through tracing the origins and genesis mechanisms of these CNVs and exploring the relative contributions of the genome stability of sperms and eggs to reproductive health, we hope to know more about genome structure variations in germ cells, enhance the effective differential diagnosis and prenatal diagnosis of genomic disorders and to provide the potential for the prevention and treatment of related diseases in the future.

## Results

### CNVs analysis and selection

**Figure 1:**
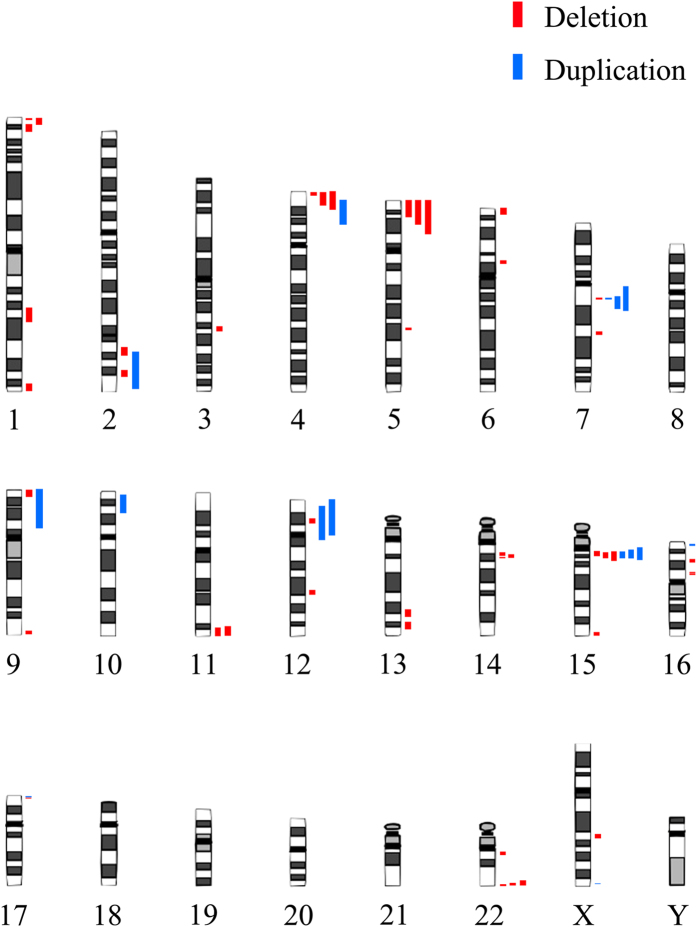
The distribution of 87 *de novo* CNVs on human chromosomes. Red bars represent deletions and blue bars represent duplications.

Using SNP arrays and fluorescence *in situ* hybridization (FISH), 87 *de novo* suspicious pathogenic CNVs, including 69 deletions and 18 duplications, were identified in total in 87 unrelated patients with ID, DD and MCA. Ages of the probands span from 6 days to 34 years old. 65 patients carried CNVs overlapping with known chromosome syndromes like Wolf-Hirschhorn syndrome (WHS), Williams Beuren syndrome (WBS) and Angelman/Prader Willi syndrome (AS/PWS). The remaining 22 patients carried rare *de novo* CNVs, such as deletion area 12p12.2-p12.1, 13q31.3-q32.1 and duplication area 9p24.3-p13.1, 10p15.3-p12.33. These regions have not been defined as a syndrome or disease-related area presently. The comprehensive information of 87 families is summarized in Supplementary Table S1 and the distribution of the CNVs on chromosome is illustrated in Fig. 1. The genome sequence coordinates based on GRCh37/hg19 assembly.

### Parent-of-origin of *de novo* pathogenic CNVs

Analysis of parental origin was performed on *de novo* pathogenic CNVs mentioned above. By means of STR profiling (69 cases) and SNP genotyping (18 cases) techniques, the parental origin of all the 87 cases were successfully determined. Among the 69 cases with deletions 41 had arisen paternally and 28 maternally (Table 1). Four types were observed in 18 cases with duplications, 10 cases were heterologous maternal allele duplication, 4 cases were homologous maternal allele duplication and 2 cases were heterologous or homologous paternal allele duplication respectively (Table 2). Two-tailed χ^2^ tests on fourfold contingency table were carried out for statistical analyses.

### Analysis of genome sequence nearby breakpoints

DNA sequences at all 87 CNVs breakpoints were preliminary analyzed in our research. According to the distribution of segmental duplications (SDs)/low-copy repeats (LCRs) at the fractured regions, CNVs were divided into the following three types: (1) SDs/LCRs existed in both ends of the CNV; (2) SDs/LCRs existed in one end of the CNV; (3) None of SDs/LCRs existed in either end of the CNV. Results of classification are shown in Supplementary Table S1.

## Discussion

As mentioned above, variations of germ line cells may arise in many stages during embryonic development like premeiotic, meiotic, or postzygotic cells. Whenever occurred, these variations would only involve germ line cells, without affecting somatic cells in the parents. Therefore, when analyzing blood samples from parents, we would not find them carrying the same pathogenic CNV as the patient. Such CNVs are classified as *de novo* in our research. Until now, systematic researches on parent-of-origin of *de novo* pathogenic CNVs were relatively few. In 2006, Thomas NS published their results on 115 *de novo* unbalanced abnormalities and indicated a pronounced paternal bias. The author thought the bias varied by chromosome and recombination patterns^13^. Later, Hehir-Kwa JY and colleagues^14^ also reported a paternal bias in 118 autosomal *de novo* CNVs discovered in 108 ID patients. The difference between them is that the latter declared the bias was independent of CNV type and length. Unlike these findings, the research from Itsara A, *et al*.^15^ showed no evidence for a parent-of-origin preference in a relatively small sample (47 *de novo* CNVs). For some familiar recurrent genomic disorders, Thomas NS, *et al*.^16^ determined the parental and chromosomal origins of several groups of patients involving 7q11.23, 15q11-q13 and 22q11, and no significant parental bias was observed except for duplications of 15q11-q13, which represented a maternal bias. And they also found a slight overall excess (just reach statistical significance, p = 0.05) of maternal bias for 22q11 deletions when combining their data with those from previous studies. This bias was then replicated and confirmed by Delio *et al*.^17^ which a total of 810 combined results revealed a highly significant maternal bias occurring in the origin of the *de novo* 22q11.2 deletion. Besides, some other specific micro-deletions/duplications have also been reported with a discrepant proportion of maternal or paternal origins, such as *NF1* region with an excess of maternal deletions^18^, the ~550 kbp autism-associated region on 16p11.2 with a maternal bias for both *de novo* deletions and duplications^19^, Sotos syndrome and 22q13 deletion syndrome with an excess of paternal deletions^20^,^21^,^22^,^23^.

**Figure 2:**
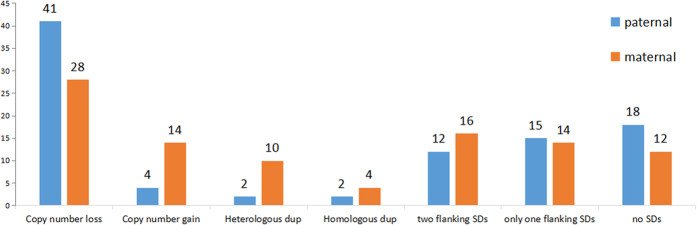
A multiple comparison between paternal CNVs and maternal CNVs. A clear difference was found when comparing the number of *de novo* CNVs according to CNV types. Paternal CNVs were noticeably more in deletions, and maternal were dominant in duplications especially in heterologous group.

In our study, the parent-of-origin of all the 87 *de novo* pathogenic CNVs were analyzed. Among them 45 (51.7%) derived from the paternal germ line and 42 (48.3%) from the maternal germ line, with no obvious bias (two-tailed binomial test, p = 0.83). However, we found a clear bias when analyzing the parental origin in different CNV types. The cases of hemizygous deletions were found to be paternal in 59.4% (41/69) and maternal in 40.6% (28/69). Meanwhile, the duplications were found to be paternal in 22.2% (4/18) and maternal up to 77.8% (14/18) (Table 3, Fig. 2). The value of chi-square test (χ^2^ = 7.911, p = 4.914 × 10^−3^) suggested that the distribution of the parent-of-origin varied significantly in different CNVs types. Combining with these data, we inferred that a paternal germline bias existed in deletions, and an evident excess of maternal origin existed in the type of duplications. For the reasons that deletions are much more likely than duplications to result in moderate-to-severe ID and major congenital anomalies, and duplications are more frequently associated with incomplete penetrance and highly variable phenotypes, it is difficult to obtain an adequate sample for duplications comparing with deletions (only 18 in our study) in this regard. For the sample size of 87, we had 95% power to detect a difference of 0.20 from the null hypothesis of 0.5. We need to point out that our observations are not a “general trend” in all *de novo* pathogenic CNVs on account of sampling bias. Our data and discussion are restricted to patients with ID, DD and MCA mentioned in Table S1.

From the perspective of the entire development process of human germline, this sex differences in parent-of-origin can be largely explained by the relative greater numbers of DNA replication and cell divisions in male than female^24^. During a male’s life, self-renewing spermatagonial stem cells continuously produce new mature sperms, while female genetic material in oogonia is already fixed before birth and will stay in early days of meiosis I for a long time until adolescence. Considering that all of 87 patients in our study were sporadic with the absence of relevant family history, and that mosaicism is rare, it can be speculated that the CNVs should mostly occur in the period of meiosis. For 87 *de novo* pathogenic CNVs with clearly parental origin, we attempted to infer their formation times. In meiosis I, primary spermatocytes and oocytes finished homologous recombination^25^. The unequal exchange between non-sister chromatids could produce deletion and heterologous allele duplication. The interchromatid breakage and exchange during the second meiosis would also produce deletion but with homologous allele duplication. So, it can be concluded that the CNV type of allele hemizygous deletion can form at any time in meiosis, heterologous allele duplications should form before the separation of homologous chromosomes in meiosis I, while homologous allele duplications might form in the interval between the separation of homologous chromosomes and the separation of sister chromatids in meiosis II. Our previous study on a patient with 2q partial trisomy presenting an inverted duplicated neocentric marker chromosome also reached consistent conclusion^26^.

Therefore, based on the replication-dependent variation in human, it is not difficult to understand the obvious paternal bias in deletions. However, if replication-based mechanisms are responsible for most *de novo* CNV formations, a paternal bias might also been observed for duplications. The maternal bias we found in duplications did not seem in line with this theory. For further details, we consulted previous publications and found there were still similar findings with a maternal bias for duplications in some specific micro-duplication syndromes like 15q11-q13 duplication syndrome^16^ and duplications in 16p11.2^19^. Furthermore, in our study, we also found difference in the degree of maternal bias between heterologous and homologous duplications, with more maternal CNVs in the heterologous duplications (Table 2). The reason would be that, as mentioned, heterologous allele duplications happened mainly in early period of meiosis I yet that is the longest residence time in the life of oocytes. One more thing should be cautioned is that our observation stands in conflict with some previous publications mentioned above which have noted maternal biases in specific micro-deletion syndromes. In two of these studies, the skewing of recombination rates between female and male was suggested to be the most likely explanation for the maternal biases in 22q11.2 and 16p11.2 regions^17^,^19^. In order to explore whether the maternal bias we found in duplications was affected by a higher female recombination rate, we examined the data from published recombination maps^27^,^28^,^29^ and calculated mean recombination rates (cM/Mb) of females and males from three studies (Marshfield, deCODE, and Genethon) in each duplication region. Higher mean female recombination rates were found in 10 (55.5%) duplications. Compared with the increase of 27.8% in detected maternal duplications, the increase of 5.5% in female-to-male recombination rates was only slightly higher for these regions. Therefore, the recombination rate could not entirely explain the observed bias in our study. Hypothesis of male-driven molecular evolution proposed by Miyata *et al*.^30^,^31^,^32^ claimed that the male germline mutations served as the major factor motivating the human molecular evolution. Nevertheless, different mechanisms participate disproportionately to various types of structural variations. Many other mechanisms may also have an influence on chromosome unbalanced recombination and the parental origin of *de novo* CNVs. The distinction at the type of recombination, the specific loci on chromosomes involved, synapsis and errors repair processes during meiotic, the severity of the phenotype, genomic imprinting, recombination rates and gender-specific recombination hotspots between males and females are all likely to produce bias on this kind of research. It is noteworthy that our study also shed light on the probable bias of paternal origin in WHS and maternal origin in 1p36 deletion syndrome and 15q11-q13 duplication syndrome, but each is small in size. Overall, current researches on the parental origin of CNVs are rare and based on different race and diseases, and even the results from various studies are inconsistent. Considering many other inevitable factors, an exact consensus remains further exploration.

**Figure 3:**
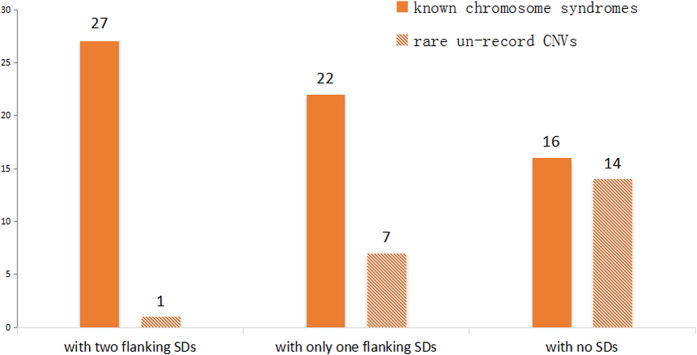
The existence of flanking SDs nearby the CNV breakpoints.

To clarify the possible formation mechanisms of the CNVs detected, we intercepted and checked the sequence at ±10 kb of both proximal and distal breakpoints, and found 57 of 87 CNVs having at least one breakpoint directly flanked by SDs. Combining the data from our study, the size of *de novo* suspicious pathogenic CNVs ranged from 0.5 Mb to 42.2 Mb, most of which were chromosome syndromes that had been well-known. Half of these syndromes often share with the similar breakpoints and rearranged fragments in unrelated individuals, called recurrent genomic disorders, such as 22q11.2 deletion syndrome, WBS and AS/PWS. As there are the same contiguous genes existed in deletion or duplication intervals, it is easier to analyze the genotype-phenotype correlations^33^. As is well known, recurrent rearrangements are mainly mediated by non-allelic homologous recombination (NAHR), the first mechanism recognized in the human genome abnormality^34^. Yet, as a common genomic architecture for these rearrangements, SDs/LCRs usually enriched near the breakpoints. In our study, the breakpoints of 49 (86%) CNVs causing well-known chromosome syndromes were clustered inside SDs, while only 8 (14%) of rare non-record *de novo* CNVs. And it can be seen from Fig. 3 that for those non-record suspicious pathogenic CNVs, nearly 2 out of 3 were not flanked by SDs in both breakpoints, and the remaining showed characteristic SDs only at one breakpoint except for one case. Besides, SDs were not attendant upon a small amount of known syndromes. Interestingly, we noticed that the breakpoints of these syndromes are scattered in the genome, some even with no common region of overlap. It just coincided well with the concept of the non-recurrent rearrangements. The absence of SDs suggested that the generation mechanism of these rearrangements was probably not NAHR but some of others like non-homologous endjoining (NHEJ), fork stalling and template switching (FoSTeS) or microhomology-mediated break-induced replication (MMBIR), in which SDs were not an indispensable requirement^35^. In portion of non-recurrent rearrangements, some shorter and highly homologous repetitive sequences such as long interspersed nuclear elements (LINEs), long terminal repeats (LTRs) and *Alus* are also functioning as substrates for NAHR as well^35^,^36^, which could explain why we found quite a number of CNVs in our study only be flanked by one SD rather than two. To our knowledge, several disorders have been proved to be non-recurrent events occurred via NHEJ, FoSTeS or MMBIR, including Miller-Dieker lissencephaly syndrome^37^, 17p13.3 duplication syndrome^38^, Pelizaeus-Merzbacher disease (PMD)^39^ and MECP2 duplication syndrome^40^. Our analysis of the sequence surrounding the breakpoints regarding these disorders also reached the same conclusion. In addition, our results of SDs/LCRs analysis nearby CNVs in different parental origins (Fig. 2) revealed that the paternal origin bias we found in deletion CNVs might have relations with mechanisms of NHEJ, FoSTeS or MMBIR, because of the higher proportion of paternal *de novo* CNVs with no SDs around. And this is also corresponding with our earlier discussion on paternal bias as these mechanisms are all replication-dependent. Of course, our analysis on sequence homology near the CNVs breakpoints was a rough estimate. The specific relationship between pathogenesis and formation mechanism of CNVs remains to be explored with precise breakpoint position mapping and related functional studies.

In summary, this is the first research on the parent-of-origin of *de novo* pathogenic CNVs of the Chinese population with ID, DD and MCA. We herein provide a clue on the significant role of human reproductive cell in the generation of *de novo* genome SVs, prompting that deletion CNVs are ordinarily paternal in origin and the relative contribution of paternal germline variations is greater in the origin of deletion CNVs, while maternal germline variations seem to be more important to duplication CNVs, especially the heterologous duplications. The analyses of the sequences flanking the breakpoints confirm that majority of recurrent CNVs arise by NAHR which relies on SDs. And non-SDs-based replication-dependent mechanisms serve as major characters in rare non-recurrent CNVs and may relate to the paternal germline bias in deletion CNVs. However, it is worth noting that our founding are not entirely consistent with what have been observed in previous literature. These discrepancies may be due to the differences in race, the composition of cohort, the design of experiment and other inevitable factors. With the rapid development of deep sequencing and bioinformatic analyses on a global genome scale, there is no doubt that we will learn more about human genomic disorders in the future and do better in the prediction and prevention of birth defects in newborns.

## Materials and Methods

### Patient samples

Clinical genetic testing for more than 1000 probands with ID, DD and MCA were completed from 2009 to 2015 at the State Key Laboratory of Medical Genetics, Central South University (Hunan, China) and Hunan Jiahui Genetics Hospital (Hunan, China). Using SNP arrays and FISH, 240 patients were found to have suspicious pathogenic CNVs. Excluding families that the parental samples were not available, a total of 87 *de novo* suspicious pathogenic CNVs identified in 87 unrelated patients in the cohort above were included in our study. DNA samples from both parents were available in all the 87 families. For each family, an informed consent was signed before testing, and then 5 ml*2 peripheral blood (EDTA anticoagulant and heparin anticoagulant respectively) were collected from the patient and the parents. The study was approved by the Ethics Committee of the state key laboratory of medical genetics of China.

### SNP array analysis

Genomic DNA of the family members was prepared from fresh peripheral blood lymphocytes using the standard phenol-chloroform method. SNP array analysis of the extracted DNA was performed using the Human OmniZhongHua-8 Beadchip or HumanCytoSNP-12 BeadChip array (Illumina Inc., San Diego, CA) following the manufacturer’s recommended protocols (www.illumina.com). The two kinds of microarrays contain about 900,000 and 300,000 probes respectively. CNVs were detected with the cnvPartition algorithm and the data including B allele frequency and log R ratio were calculated using GenomeStudio software (cnvPartition Plugin v3.1.6). We picked out CNVs with a call rate >0.99 and a confidence >35 for further analyses.

### CNVs identification and analysis

FISH analysis was performed on metaphase or interphase chromosomes from the patients and their parents to determine the authenticity and origin of CNVs. For each CNV, two BAC clone probes with different fluorescence were used. One located within the deletion or dupliction region as a test probe, and another located within normal region in the same chromosome as a control probe. Alternatively, for small CNVs which were less than 1 Mb and hardly identified by regular FISH analysis, SNP Array analysis of the parents’ DNA was utilized. Judgment of the pathogenicity of *de novo* CNVs has always been an intractable thing for clinical staffs and geneticist. Following the recommendations for systematic evaluation and clinical interpretation of CNVs from Miller *et al*. and American College of Medical Genetics (ACMG)^41^,^42^, we considered about various characteristics including CNV size, origin, inheritance mode and genomic content (including genes, exons and regulatory elements) in the CNV interval, and referenced latest published literature and publicly external databases such as OMIM (http://omim.org/), DECIPHER (https://decipher.sanger.ac.uk/), ISCA (http://dbsearch.clinicalgenome.org/search/) and DGV (http://dgv.tcag.ca/dgv/app/home). Ultimately, only *de novo* suspicious pathogenic CNVs were picked out according to the criteria or features below: (1) The CNV overlaps a critical interval with well-characterized genomic disorders (even if inherited from normal parents); (2) The CNV region contains haploinsufficient or triple sensitive diseases-associated genes; (3) Size of the CNV is large (generally >1 Mb, but also consider gene content) and it is not been observed in general population, especially *de novo*; (4) A similar CNV has been studied in large cohort showing significant association with a defined phenotype; (5) The CNV is recurrent in patients but lack of statistical power temporarily due to its rarity; (6) Deletion in a gene may cause disease by loss of heterozygosity (LOH).

### STR profiling/SNP genotyping

Two approaches were used to estimate parental origin of *de novo* suspicious pathogenic CNVs detected in the patients. For STR profiling, proper microsatellites within the deleted or duplicated region were selected. Information about the specific location, heterozygosity in the crowd, primers and range of variation of microsatellites were taken from Marshfield Genetic Maps (http://www.bli.uzh.ch/BLI/Projects/genetics/maps/marsh.html) and UCSC Genome Browser (http://genome.ucsc.edu/). Analysis of markers was conducted on ABI 3100 Genetic Analyzer. By analyzing the different peaks of the same microsatellite loci among the proband and the parents in each pedigree, we obtained relevant information on the origin of CNVs. The second method based on SNPs was applied for CNVs without encompassing informative microsatellite loci. We defined the parent-of-origin by comparing the difference between the SNP loci in family members with two assisting methods: Sanger sequencing of the genes located in the interval of SNP-rich CNVs or analyzing the genotyping calls and B allele frequency in each CNV regions exported via GenomeStudio software. In general, we identified the parental origin based on at least two informative STR or SNP loci, whereas in a handful of cases only one was informative. All the methods were carried out in accordance with the approved guidelines.

### Structural analysis of genome sequence near the CNVs breakpoints

To investigate the sequence homology near the CNVs breakpoints and reveal possible mechanisms of the disease-related CNVs formation, the genome sequence within a 20 kb window(±10 kb in either end) of all the 87 CNVs breakpoints were analyzed.

## Additional Information

**How to cite this article**: Ma, R. *et al*. A clear bias in parental origin of *de novo* pathogenic CNVs related to intellectual disability, developmental delay and multiple congenital anomalies. *Sci. Rep.* **7**, 44446; doi: 10.1038/srep44446 (2017).

**Publisher's note:** Springer Nature remains neutral with regard to jurisdictional claims in published maps and institutional affiliations.

## Supplementary Material

Supplementary Information

## Figures and Tables

**Table 1:** STR fragment size and SNP genotyping results of *de novo* deletion CNVs.

Paternal hemizygous deletion				Maternal hemizygous deletion			
Patient ID	Patient	Father	Mother	Patient ID	Patient	Father	Mother
MD1459	**187**	189/191	185/**187**	MD1908	**92**	89/**92**	79/79
MD1506	**410**	393/430	**410**/418	MD2484	**100**	92/**100**	92/92
MD1672	**199**	191/207	183/**199**	MD2586	**136**	**136**/140	140/143
MD1975	**238**	221/226	226/**238**	MD3164	**116**	105/**116**	105/118
MD2389	**270**	268/272	**270**/272	MD3778	**C**	**C**/T	T/T
MD2750	**100**	117/119	**100**/117	MD4612	**144**	**144**/160	152/168
MD2994	**146**	150/150	**146**/150	MD5599	**283**	268/**283**	259/268
MD4350	**120**	136/136	115/**120**	MD6474	**170**	167/**170**	167/167
MD4748	**205**	209/211	**205**/207	MD6894	**143**	**143**/147	150/159
MD5621	**151**	148/155	**151**/155	MD8177	**A**	**A**/A	C/C
MD6227	**204**	197/206	**204**/212	MD8557	**262**	**262**/300	254/303
MD6723	**222**	216/224	216/**222**	MD8877	**115**	101/**115**	101/101
MD7132	**124**	120/130	**124**/126	MD9792	**209**	195/**209**	205/205
MD7454	**287**	283/295	275/**287**	MD10290	**155**	**155**/159	143/151
MD8160	**A**	G/G	**A**/A	MD10369	**283**	**283**/287	280/287
MD8417	**254**	226/256	**254**/267	MD11049	**169**	**169**/175	164/167
MD9127	**A**	G/G	**A**/G	MD11905	**285**	262/**285**	262/297
MD9478	**146**	132/144	132/**146**	MD12173	**155**	151/**155**	151/159
MD11042	**163**	161/161	161/**163**	MD12563	**293**	262/**293**	266/289
MD11193	**131**	129/137	**131**/131	MD12665	**95**	89/**95**	91/93
MD11326	**140**	123/137	136/**140**	MD13120	**212**	208/**212**	208/210
MD11345	**G**	C/C	**G**/C	MD13380	**146**	**146**/150	142/150
MD11473	**G**	C/C	**G**/G	MD14271	**95**	**95**/97	92/92
MD11966	**A**	C/C	**A**/C	MD14442	**A**	**A**/C	C/C
MD11973	**C**	A/A	A/**C**	MD14816	**267**	263/**267**	259/271
MD12285	**A**	G/G	**A**/G	MD15823	**262**	258/**262**	258/266
MD12779	**C**	A	A/**C**	MD16014	**132**	129/**132**	130/134
MD12807	**226**	248/261	**226**/230	MD16085	**G**	**G**/G	C/C
MD12812	**266**	242/262	**266**/266				
MD13097	**272**	266/274	262/**272**				
MD13108	**150**	158/164	**150**/164				
MD13636	**166**	154/158	164/**166**				
MD14047	**147**	133/139	133/**147**				
MD14491	**139**	137/143	**139**/141				
MD14517	**A**	G/G	**A**/G				
MD14650	**121**	114/119	**121**/125				
MD15641	**144**	138/138	138/**144**				
MD15761	**G**	A/A	A/**G**				
MD16107	**127**	122/122	113/**127**				
MD16204	**A**	G/G	**A**/G				
MD16619	**C**	A/A	A/**C**				

The genotyping data from one of informative markers was listed.The bold font indicates the same STR/SNP locus between patients and their parents.

**Table 2:** STR fragment size and SNP genotyping results of *de novo* duplication CNVs.

Homologous maternal allele duplication				Heterologous maternal allele duplication			
Patient ID	Patient	Father	Mother	Patient ID	Patient	Father	Mother
MD11928	A/**C/C**	A/A	A/**C**	MD1643	**268**/**275**/301	260/301	**268**/**275**
MD12320	270/**289/289**	270/285	262/**289**	MD4267	**185**/**187/**195	193/195	**185**/**187**
MD13935^*^	**G/G**	A	A/**G**	MD4278	**130**/**132**/134	122/134	**130**/**132**
MD15100	**181/181**/190	185/190	**181**/181	MD4477	**162**/167/**173**	167/179	**162**/**173**
				MD7381	**254**/**258**/262	262/266	**254**/**258**
				MD7484	**135**/**147**/151	151/155	**135**/**147**
				MD8152	**228/247**/264	256/264	**228**/**247**
				MD13015	107/**117/121**	107/111	**117/121**
				MD15558	259/**263/298**	259/282	**263/298**
				MD15714	**134**/136/**138**	136/148	**134/138**
**Homologous paternal allele duplication**				**Heterologous paternal allele duplication**			
**Patient ID**	**Patient**	**Father**	**Mother**	**Patient ID**	**Patient**	**Father**	**Mother**
MD10790	161/**186/186**	174/**186**	161/194	MD4571	**106**/**115**/140	**106**/**115**	125/140
MD15000	205/**225/225**	220/**225**	205/234	MD8294	**165**/**182**/194	**165**/**182**	178/194

The genotyping data from one of informative markers was listed. The bold font indicates the same STR/SNP locus between patients and their parents.

*The patient is a male with a 0.5 Mb duplication on Xq28. The SNP loci within the region of Xq28 showed homozygous G from the mother which indicated a maternal allele duplication.

**Table 3:** The distribution of the parent-of-origin in different CNVs types.

	Parent-of-origin		Total number	Two-Tailed p value*
	Paternal	Maternal		
Deletions	41	28	69	4.914 × 10^−3^
Duplications	4	14	18	
Heterologous	2	10	12	
Homologous	2	4	6	
Total number of CNVs	45	42	87	

*Chi-square test using IBM SPSS Statistics 20.0.
