# Supplementary material for: A clear bias in parental origin of de novo pathogenic CNVs related to intellectual disability, developmental delay and multiple congenital anomalies
Source: Sci Rep. 2017 Mar 21;7:44446. doi: 10.1038/srep44446 (PMC5359547; doi:10.1038/srep44446)
Supplement: Supplementary Information [file srep44446-s1.doc]

**Supplementary Information**

**A clear bias in parental origin of *de novo* pathogenic CNVs related to intellectual disability, developmental delay and multiple congenital anomalies**

Ruiyu Ma^1^, Linbei Deng^1^, Yan Xia^1^, Xianda Wei^1^, Yingxi Cao^1^, Ruolan Guo^1^, Rui Zhang^1^, Jing Guo^1^, Desheng Liang^1,*^, Lingqian Wu^1,*^

^1^State Key Laboratory of Medical Genetics, Central South University, Changsha, Hunan 410078, P.R. China

*Desheng Liang, M.D., Ph.D. liangdesheng@sklmg.edu.cn

*Lingqian Wu, M.D., Ph.D. [wulingqian@sklmg.edu.cn](mailto:wulingqian@sklmg.edu.cn)

**Corresponding author:**

Desheng Liang, M.D., Ph.D. or Lingqian Wu, M.D., Ph.D., State Key Laboratory of Medical Genetics, Central South University, 110 Xiangya Road,Changsha, Hunan 410078, China

Tel.: +86 731 84805252

Fax: +86 731 84478152

E-mail: [liangdesheng@sklmg.edu.cn](mailto:liangdesheng@sklmg.edu.cn) (Desheng Liang, M.D., Ph.D.) or

[wulingqian@sklmg.edu.cn](mailto:wulingqian@sklmg.edu.cn) (Lingqian Wu, M.D., Ph.D.)

Supplementary Table S1 The comprehensive information of 87 pedigrees with congenital birth defects

| **No.** | **Patient ID** | **Age** | **Gender** | **Complaint** | **Cytoband** | **Size (Mb)** | **Type** | **Overlapping with known syndromes** | **Category** |
| --- | --- | --- | --- | --- | --- | --- | --- | --- | --- |
| 1 | MD1459 | 4y | M | DD | 13q31.3-q32.1 | 8.0 | Del |  | (3) |
| 2 | MD1506 | 6y | F | ID | 14q11.2-q12 | 2.5 | Del |  | (2) |
| 3 | MD1643 | 9y | M | ID | 15q11.1-q13.2 | 11.2 | Dup | 15q11-q13 duplication syndrome | (3) |
| 4 | MD1672 | 12y | M | ID | 12q21.31 | 3.6 | Del |  | (3) |
| 5 | MD1908 | 2y | M | MCA | 5q23.1-q23.2 | 2.0 | Del |  | (3) |
| 6 | MD1975 | 1y | F | DD | 3q22.3-q23 | 4.2 | Del | BPES | (3) |
| 7 | MD2389 | 10y | M | cleidocranial dysplasia | 6p21.1-p12.3 | 3.5 | Del | Cleidocranial dysplasia | (3) |
| 8 | MD2484 | 11y | M | ID | 7q11.23 | 1.4 | Del | WBS | (2) |
| 9 | MD2586 | 2y | M | DD | 12p12.2-p12.1 | 4.4 | Del |  | (3) |
| 10 | MD2750 | 9y | F | Motor Delay | 7q11.23 | 1.4 | Del | WBS | (2) |
| 11 | MD2994 | 1m | M | Poor response | 15q11.2-q12 | 5.0 | Del | PWS | (2) |
| 12 | MD3164 | 2m | M | Lissencephaly | 17p13.3 | 0.6 | Del | Miller-Dieker lissencephaly syndrome | (3) |
| 13 | MD3778 | 6y | F | Delayed speech and language development | 1p36.33-p36.32 | 1.6 | Del | 1p36 deletion syndrome | (2) |
| 14 | MD4267 | 2y | M | DD | 9p24.3-p13.1 | 38.7 | Dup |  | (2) |
| 15 | MD4278 | 5y | M | ID | 7q11.1-q21.12 | 25.4 | Dup |  | (2) |
| 16 | MD4350 | 2m | F | DD | 4p16.3-p15.31 | 18.6 | Del | WHS | (2) |
| 17 | MD4477 | 5y | M | Delayed speech and language development | 16p13.3 | 1.6 | Dup | 16p13.3 duplication syndrome | (2) |
| 18 | MD4571 | 9y | M | ID | 10p15.3-p12.33 | 18.1 | Dup |  | (2) |
| 19 | MD4612 | 1y | F | ID | 13q33.1-q34 | 8.1 | Del |  | (3) |
| 20 | MD4748 | 9y | M | ID | 2q36.2-q37.1 | 5.8 | Del |  | (3) |
| 21 | MD5599 | 1y | F | DD | 15q11.2-q13.1 | 6.3 | Del | AS | (1) |
| 22 | MD5621 | 3y | M | Cerebral white matter dysplasia | 15q11.2-q13.1 | 5.8 | Del | PWS | (1) |
| 23 | MD6227 | 1y | F | DD | 14q12 | 0.8 | Del | Rett syndrome | (3) |
| 24 | MD6474 | 2m | M | CHD | 1q43-q44 | 8.8 | Del | 1q43-q44 deletion syndrome | (3) |
| 25 | MD6723 | 8y | M | DD | 1q24.3-q31.1 | 16.4 | Del |  | (3) |
| 26 | MD6894 | 1y | M | DD | 15q11.2-q13.1 | 7.0 | Del | AS | (2) |
| 27 | MD7132 | 1y | M | MR | 9p24.3-p24.1 | 7.6 | Del | monosomy 9p deletion syndrome | (2) |
| 28 | MD7381 | 7m | F | DD | 15q11.1-q13.1 | 8.9 | Dup | 15q11-q13 duplication syndrome | (1) |
| 29 | MD7454 | 8y | F | DD, ID | 22q13.32-q13.33 | 2.1 | Del | Phelan-McDermid syndrome | (2) |
| 30 | MD7484 | 3y | F | ID, DD | 15q11.1-q13.3 | 12.4 | Dup | 15q11-q13 duplication syndrome | (1) |
| 31 | MD8152 | 6d | M | MCA | 12p13.33-p11.1 | 34.6 | Dup | PKS | (3) |
| 32 | MD8160 | 4y | F | ID | 22q11.21 | 2.6 | Del | 22q11.2 deletion syndrome | (1) |
| 33 | MD8177 | 8y | M | ID | 7q11.23 | 1.4 | Del | WBS | (2) |
| 34 | MD8294 | 1y | M | DD | 7q11.22-q21.11 | 12.9 | Dup | 7q11.23 duplication syndrome | (3) |
| 35 | MD8417 | 1y | F | MCA | 5p15.33-p14.2 | 23.8 | Del | cri-du-chat syndrome | (2) |
| 36 | MD8557 | 34y | M | ID | 1p36.22-36.13 | 7.6 | Del | 1p36 deletion syndrome | (3) |
| 37 | MD8877 | 3y | M | ID | 15q11.2-q13.1 | 4.9 | Del | AS | (1) |
| 38 | MD9127 | 4y | M | MR, EP | 22q11.21 | 2.6 | Del | 22q11.2 deletion syndrome | (1) |
| 39 | MD9478 | 4y | F | ID, Obesity | 15q11.2-q13.1 | 4.9 | Del | PWS | (1) |
| 40 | MD9792 | 2m | F | CHD | 5p15.33-p15.1 | 17.6 | Del | cri-du-chat syndrome | (2) |
| 41 | MD10290 | 5y | M | ID, Paroxysmal laughter | 15q11.2-q13.1 | 5.8 | Del | AS | (2) |
| 42 | MD10369 | 2y | M | MR | 22q13.33 | 1.7 | Del | Phelan-McDermid syndrome | (3) |
| 43 | MD10790 | 2y | F | DD, Distinctive facial features | 2q33.3-2q37.3 | 34.3 | Dup |  | (2) |
| 44 | MD11042 | 6y | F | MR, Microcephaly, Behavioral abnormality | 2q33.1-2q34 | 8.9 | Del |  | (3) |
| 45 | MD11049 | 7y | F | MR，Delayed speech and language development | 1q43-q44 | 7.1 | Del | 1q43-q44 deletion syndrome | (2) |
| 46 | MD11193 | 2m | F | MCA | 9q34.3 | 3.5 | Del | Kleefstra syndrome | (2) |
| 47 | MD11326 | 6y | M | Learning disabilities | 16p13.11-12.3 | 2.7 | Del |  | (3) |
| 48 | MD11345 | 4y | M | DD, CHD, Deafness, Autism | 22q11.21 | 2.6 | Del | 22q11.2 deletion syndrome | (1) |
| 49 | MD11473 | 4y | M | CHD, Delayed speech and language development | 22q11.21 | 2.6 | Del | 22q11.2 deletion syndrome | (1) |
| 50 | MD11905 | 2y | F | Family history of AS | 15q11.2-q13.1 | 6.5 | Del | AS | (1) |
| 51 | MD11928 | 8y | M | MR | 17p13.3 | 0.8 | Dup | 17p13.3 duplication syndrome | (3) |
| 52 | MD11966 | 16y | M | DD, CHD, Cleft palat | 22q11.21 | 2.6 | Del | 22q11.2 deletion syndrome | (1) |
| 53 | MD11973 | 4y | F | DD | 14q12 | 2.8 | Del | Rett syndrome | (3) |
| 54 | MD12173 | 3y | M | EP, Paroxysmal laughter | 15q11.2-q13.1 | 4.9 | Del | AS | (1) |
| 55 | MD12285 | 28y | M | ID, Distinctive facial features | 22q11.21 | 2.6 | Del | 22q11.2 deletion syndrome | (1) |
| 56 | MD12320 | 4y | M | MR | 15q11.2-q13.1 | 5.4 | Dup | 15q11-q13 duplication syndrome | (1) |
| 57 | MD12563 | 3y | M | DD, EP, Paroxysmal laughter | 15q11.2-q13.1 | 4.9 | Del | AS | (1) |
| 58 | MD12665 | 2y | M | MR | 7q11.23 | 1.4 | Del | WBS | (1) |
| 59 | MD12779 | 10y | F | ID, Delayed speech and language development, Early menarche | Xq22.1-q22.3 | 3.9 | Del |  | (3) |
| 60 | MD12807 | 10y | F | Mild ID, Short stature | 11q24.2-q25 | 10.0 | Del |  | (3) |
| 61 | MD12812 | 4m | M | DD | 15q11.2-q13.1 | 5.8 | Del | PWS | (1) |
| 62 | MD13015 | - | F | ID | 7q11.23 | 1.3 | Dup | 7q11.23 duplication syndrome | (2) |
| 63 | MD13097 | 12y | F | CHD, Mild ID | 11q24.2-q25 | 8.9 | Del | Jacobsen syndrome | (3) |
| 64 | MD13108 | 8m | F | DD | 5p15.33-p14.2 | 23.7 | Del | cri-du-chat syndrome | (2) |
| 65 | MD13120 | 7m | F | DD | 15q26.3 | 3.2 | Del | 15q26-qter deletion syndrome | (2) |
| 66 | MD13380 | 4y | F | ID, Paroxysmal laughter | 15q11.2-q13.1 | 4.9 | Del | AS | (1) |
| 67 | MD13636 | 7m | F | DD | 6p25.3-p25.1 | 5.8 | Del |  | (2) |
| 68 | MD13935 | 6y | M | DD | Xq28 | 0.5 | Dup | MECP2 duplication syndrome | (2) |
| 69 | MD14047 | 4y | M | EP, MR, MCA | 4p16.3-p15.33 | 12.7 | Del | WHS | (3) |
| 70 | MD14271 | 4y | F | MR | 5p15.33-p13.3 | 33.1 | Del | cri-du-chat syndrome | (2) |
| 71 | MD14442 | 8y | F | Delayed speech and language development, EP, Gait irregularity,  Hyperexplexia,  Flapping movement | 15q11.2-q13.3 | 9.8 | Del | AS  15q11.2 deletion syndrome  15q13.3 microdeletion syndrome | (1) |
| 72 | MD14491 | 7y | M | MR | 15q11.2-q13.1 | 6.6 | Del | PWS | (1) |
| 73 | MD14517 | 3y | F | MR, EP | 16p11.2 | 1.0 | Del |  | (2) |
| 74 | MD14650 | 4y | F | Delayed speech and language development, Motor delay | 22q13.31-q13.33 | 4.2 | Del | Phelan-McDermid syndrome | (2) |
| 75 | MD14816 | 4y | M | DD, EP, Paroxysmal laughter | 15q11.2-q13.1 | 4.9 | Del | AS | (1) |
| 76 | MD15000 | 4m | F | Mild ID, CHD, Chromosomal anomaly | 11p15.5-p15.4 | 8.7 | Dup |  | (3) |
| 77 | MD15100 | 10m | F | DD | 12p13.31-q12 | 33.0 | Dup |  | (3) |
| 78 | MD15558 | 8y | M | Mild ID, Autistic behavior | 15q11.1-q13.1 | 8.9 | Dup | 15q11-q13 duplication syndrome | (1) |
| 79 | MD15641 | 2y | F | DD | 5p15.33-p15.1 | 17.8 | Del | cri-du-chat syndrome | (2) |
| 80 | MD15714 | 10y | M | Mild MR, Hyperkinetic behavior | 4p16.1-p15.1 | 26.0 | Dup |  | (1) |
| 81 | MD15761 | 11y | F | MR, EP | 4p16.3 | 3.5 | Del | WHS | (3) |
| 82 | MD15823 | 5y | M | MR, EP, Paroxysmal laughter | 15q11.2-q13.1 | 4.9 | Del | AS | (1) |
| 83 | MD16014 | 5y | F | MR, EP | 1p36.32-p36.23 | 5.4 | Del | 1p36 deletion syndrome | (3) |
| 84 | MD16085 | 7y | M | MCA, EP | 22q11.21 | 2.8 | Del | 22q11.2 deletion syndrome | (1) |
| 85 | MD16107 | 1y | F | Delayed speech and language development, Motor delay | 7q22.2-q22.3 | 2.5 | Del |  | (3) |
| 86 | MD16204 | 4m | M | EP | 16p11.2 | 0.6 | Del | 16p11.2 deletion syndrome | (1) |
| 87 | MD16619 | 11m | F | DD | 15q11.2-q13.1 | 4.9 | Del | PWS | (1) |

y, year; m, month; d, day; M, male; F, female; DD, developmental delay; ID, intellectual disability; MCA, multiple congenital anomalies; CHD, [congenital](javascript:void(0);) [heart](javascript:void(0);) [disease](javascript:void(0);); MR, mental retardation; EP, epilepsy; Del, deletion; Dup, duplication; BPES, blepharophimosis, ptosis, and epicanthus inversus syndrome; WBS, Williams-Beuren syndrome; PWS, Prader-Willi Syndrome; WHS, Wolf-Hirschhorn syndrome; AS, Angelman Syndrome; PKS, Pallister-Killian syndrome; -, unknown.
